# Recent Advances in Epidural Analgesia

**DOI:** 10.1155/2012/309219

**Published:** 2011-11-24

**Authors:** Maria Bauer, John E. George, John Seif, Ehab Farag

**Affiliations:** ^1^Department of Outcomes Research, Cleveland Clinic, Cleveland, OH 44195, USA; ^2^Anesthesiology Institute, Cleveland Clinic, Cleveland, OH 44195, USA

## Abstract

Neuraxial anesthesia is a term that denotes all forms of central blocks, involving the spinal, epidural, and caudal spaces. Epidural anesthesia is a versatile technique widely used in anesthetic practice. Its potential to decrease postoperative morbidity and mortality has been demonstrated by numerous studies. To maximize its perioperative benefits while minimizing potential adverse outcomes, the knowledge of factors affecting successful block placement is essential. This paper will provide an overview of the pertinent anatomical, pharmacological, immunological, and technical aspects of epidural anesthesia in both adult and pediatric populations and will discuss the recent advances, the related rare but potentially devastating complications, and the current recommendations for the use of anticoagulants in the setting of neuraxial block placement.

## 1. Introduction

Neuraxial anesthesia is the term for central blocks involving the spinal, epidural, and caudal spaces. While it is now an invaluable adjunct and even occasionally an alternative to general anesthesia, its use is not a new phenomenon. Physicians such as Corning published studies documenting success with neuraxial blocks as early as 1885 [1]. Even more ambitious physician-scientists such as Bier became knowledgeable about spinal anesthesia, in particular, through self-investigation [2]. It unfortunately was also through this type of dedication that he became all too familiar with postdural puncture headaches. Despite its early use, though, much of the gains we have with neuraxial blocks did not occur until the early 1900's. Limitations in this particular area of anesthesia were limited to lack of drug diversity and a lack of adequate equipment. Prior to 1904, the only drug available for neuraxial use was cocaine, and development of epidural technology was still a ways off. With a larger drug base and equipment advancements came an expansion of the role of neuraxial anesthesia in anesthesia practice.

 Excluding the obvious fact that surgical conditions primarily dictate the type of anesthesia performed, most operations below the neck can be performed under neuraxial anesthesia. Various studies have shown a decrease in postoperative morbidity and even mortality when used either with general anesthesia or alone. Neuraxial blocks have even been shown to reduce the incidence of venous thrombosis and pulmonary embolism while also minimizing transfusion requirements and respiratory compromise following thoracic and upper abdominal surgery. A decreased stress response has also been noted which may have positive cardiac benefits such as reduced perioperative and postoperative ischemia. Despite these proposed advantages of neuraxial blocks, adverse reactions and complications can occur. These can range from self-limited back soreness to permanent neurologic deficits and even death. Because an expansive review of neuraxial blocks is beyond the scope of this review, we have chosen to focus our discussion to epidural and caudal anesthesia. In doing so, we will review pertinent epidural knowledge, and present cutting edge advances specific to epidural and caudal anesthesia.

## 2. Anatomy for Epidural Placement

The anatomy for the placement of an epidural goes beyond the epidural space itself. It is for this reason that this section will not only cover anatomy of this space, but also important surrounding anatomy.

The epidural space extends from the base of the skull to the sacral hiatus. Its lateral boundaries are the vertebral pedicles, while the anterior and posterior boundaries are the dura mater and ligamentum flavum, respectively. The contents of the space include fat, lymphatics, and veins with nerve roots that cross it. Determinants of epidural fat include age and body habitus with obese patients having the greatest amount of epidural fat [2]. The amount of epidural fat within the space is just one of the factors that determine volume necessary for adequate anesthesia or analgesia.

Veins within the epidural space form a plexus called Batson's venous plexus. These veins connect with the iliac and azygos veins and are significant because of a lack of valves commonly found in veins. It is the lack of these valves in conjunction with a compressed inferior vena cava from a gravid uterus, which results in the venous engorgement of epidural veins found in parturients.

Traditional thought on epidural anatomy was that it is one continuous space. A more recent thought is the concept of it being a potential space with septations or crevices formed by layering of epidural contents (fat). The anatomic layering and texture of epidural contents create inconsistent paths that ultimately make flow through it less uniform [3]. The idea of these septations or crevices forming variable paths for the flow of a solution is the rationale given for unilateral or partial epidural blockade [4].

 Vertebral spinous processes help define the midline. In the cervical and lumbar areas they are horizontal, while in the thoracic vertebrae (specifically T4 through T9) they are caudally angulated. The space between these caudally angulated spinous processes are often difficult to access leading some to favor a paramedian approach to thoracic epidural placement as opposed to the traditional midline approach. While the surgical site dictates the level of the epidural placement, the safest location is one whereby inadvertent spinal cord damage can be avoided. In adults, the spinal cord typically ends at the lower border of the L1 vertebra while in children it is at the level of the lower border of L3. By the age of 8 years, one can safely target the same lumbar levels for safe epidural placement as in the adult, while under the age of 7 years, a caudal approach to the epidural space is safest. One generally accepted landmark for assessing lumbar level for epidural placement is the superior aspect of the iliac crest. A horizontal line drawn between the superior borders of either iliac crest corresponds to the L4 vertebral body or the L4-5 interspace. For thoracic epidural placement, the inferior border of the scapula is the usual site of the T7 vertebral body/spinous process, and is typically used to approximate thoracic level of epidural placement for thoracic or intraabdominal surgical procedures. The approximate distance from the skin to the epidural space in 80% of individuals is 4–6 cm with the caveat that thin and obese patients may vary outside of this range [5].

## 3. Choices for Epidural Infusions

Local anesthetics are the mainstay of therapy for obtaining analgesia or anesthesia with an epidural. Understanding the pharmacology of local anesthetics is therefore paramount. Specifically, factors such as surgical location and duration, desire to have a sensory and/or motor block, or the expected potency and duration of a specific local anesthetic agent should be considered prior to placing an epidural block. The choice of which local anesthetic agent to use can be categorized based on desired length of action. Regardless of the class of local anesthetic, these drugs can be divided into ones that are short, intermediate, or long acting. The shortest-acting local anesthetic agent is chloroprocaine. Its short length provides ample anesthesia for short surgical procedures, and its quick elimination obviates the need for prolonged recovery room discharges.

 Lidocaine has traditionally been the agent of choice for slightly longer surgical procedures that require an intermediate-acting local anesthetic. In place of lidocaine, some centers have also adopted the use of mepivacaine for its longer length of action with a similar onset profile. The intermediate length of action of either agent can be prolonged by the addition of epinephrine. Of note is the potential for an increased incidence of hypotension due to venous pooling from the beta effects of epinephrine containing solutions. This phenomenon seems to be especially true of patients receiving lumbar epidural analgesia. 

 Longer-acting local anesthetics used for epidural blockade typically consist of either bupivacaine or ropivacaine in varying concentrations. Greater concentrations of either will produce a greater motor block in addition to the sensory block that is typically desired. Ropivacaine, an analog of mepivacaine, has a lesser intense and shorter duration of motor block in addition to a lower toxicity profile than an equipotent dose of bupivacaine [6]. The cardiac toxicity profile of bupivacaine is the highest among all the choices of local anesthetics. It is due to a high degree of protein binding and a greater blocking effect on cardiac sodium channels. 

 Multiple attempts have been made to find various additives to improve the onset and duration of an epidural block. Alkalinization with sodium bicarbonate has proven effective in a dose of 1 mEq/10 mL local anesthetic for chloroprocaine, lidocaine, or mepivacaine. A lower concentration of sodium bicarbonate (0.1 mEq/10 mL of local anesthetic) is necessary for bupivacaine and ropivacaine due to the potential of precipitation with higher concentrations. The addition of epinephrine to a local anesthetic increases the duration of action by decreasing the vascular absorption. While this phenomenon has been shown to be true with the short- and intermediate-acting local anesthetic agents, it appears to be less effective with longer-acting agents. With the low doses typically used in the epidural space, the overall cardiovascular response seems to be vasodilation (causing a decrease in mean arterial pressure), in addition to an increase in heart rate and contractility. These effects ultimately result in maintenance of cardiac output. Phenylephrine has also been used to prolong the effects of neuraxial local anesthetics. In contrast to the use of epinephrine in the epidural space, it causes an increase in peripheral vascular resistance without the added benefits of an increase in chronotropy or contractility. The resulting drop in cardiac output is the reason most anesthesiologists avoid phenylephrine in the epidural space.

 Opioids remain the analgesic adjuvant of choice for augmenting the effects of local anesthetics in the epidural space. Epidural administration of fentanyl intraoperatively has been shown to significantly reduce volatile agent requirements by more than twofold in some instances [7]. Despite the benefits of neuraxial opioids, side effects do occur. Some of the more common side effects are pruritus (specifically in the mid-facial area), nausea, and urinary retention. Hypotension can also occur which is attributed to the reduction of sympathetic outflow via opioid receptors in the sympathetic ganglia. 

 Another class of analgesic adjuvants includes alpha-adrenergic agonists. Clonidine is the main drug used in this class due to its production as a preservative-free preparation. The effects of epidurally administered clonidine are seen as early as 20 minutes after injection, with peak effects occurring in 1 hour. The analgesic potency has been described as being comparable to epidurally administered morphine [8]. Adding clonidine to opioids in the epidural space has an additive effect, which results in a lower dose of narcotic necessary for optimal pain control. This as a consequence diminishes the incidence of respiratory depression that potentially occurs with neuraxial opioids. Clonidine is lipophilic, and as a result is quickly redistributed systemically despite neuraxial injection. It therefore has both central and peripheral effects. At lower doses, the central effects cause sympatholysis leading to hypotension, while the peripheral effects at higher doses cause vasoconstriction. Clonidine administered in the low thoracic or lumbar region typically produces blood pressure effects similar to that seen with intravenous administration [9]. When given in the mid or upper thoracic regions, epidurally administered clonidine causes an even greater decrease in blood pressure [10]. This more substantial drop in blood pressure is attributed to blocking thoracic dermatomes that contribute to sympathetic fibers innervating the heart. In addition to the hypotensive potential of clonidine, bradycardia, and nausea with or without vomiting are also potential side effects. The cause of bradycardia is twofold. Clonidine has vagomimetic effects in addition to inhibiting norepinephrine release. Additional side effects such as sedation and dry mouth are possible, but seem to be dose related. Even more esoteric compounds such as neostigmine, ketamine, ketorolac, midazolam, and dexamethasone are being studied with hopes to develop additional tools to supplement or even replace the neuraxial analgesia and anesthesia of local anesthetics. While this discussion focuses on epidural use of these agents, their clinical use may have far greater application. Current studies are not only investigating these agents in the acute pain setting, but are also for use in various chronic pain disorders.

## 4. The Effect of Anesthetic Technique on Immune Function

Surgery is associated with a wide range of metabolic, endocrine, hematological, and inflammatory/immunological responses, known collectively as surgical stress response. Surgical stress response has been identified as a major factor accounting for perioperative immune suppression [11]. The extent to which this adaptive response can be modified appears to be dependent on the anesthetic and analgesic technique used, and with regards to postoperative outcomes, has been extensively studied [12, 13]. There is evidence that regional anesthesia, particularly epidural blockade, attenuates or inhibits surgical stress by blocking afferent neural stimuli from reaching the central nervous system, as well as by blocking the efferent activation of the sympathetic nervous system [14, 15]. The nervous system accounts for the main common pathway mediating the surgical stress response [16]. Immune response is subject to neuroendocrine regulation and elicits neuroendocrine changes [17], augmenting or blunting the neuroendocrine response. It therefore affects postoperative immune function, and ultimately long-term outcomes [18, 19].

## 5. Perioperative Immunosuppression and the Impact of Anesthetic Technique on Postoperative Outcomes

Impaired perioperative immunity is related to the neuroendocrine stress response. Evidence suggests that the factors that are associated with immunosuppression during surgery are surgical stress response, general anesthesia, and opioid analgesia.

 Surgical trauma in itself induces the release of catecholamines, adrenocorticotropic hormone, and cortisol, depresses cell-mediated immune responses including natural killer cell and cytotoxic T-cell function [13, 19–21], and promotes tumor vascularization [22, 23]. Additionally, risk factors, such as pain [24], blood transfusion [25], hypothermia [26], and hyperglycemia [27], further impair immunity. Pain activates the HPA axis, and may induce accelerated lymphocyte apoptosis [28]. Hypothermia impairs neutrophil oxidative killing by causing thermoregulatory vasoconstriction and thus decreasing oxygen supply [29]. Perioperative hyperglycemia impairs phagocytic activity and oxidative burst, as there is less NADPH available due to the activation of the NADPH consuming polyol pathway [30–32]. Earlier studies suggested that cell-mediated immune function [33, 34] is reduced by allogenic blood transfusion. Transfusion has more recently been suggested to facilitate host T_h_2 cells to produce immunosuppressive IL-4 and IL-10; however, the exact mechanism of causality is yet unclear [25].

 General anesthesia is also considered to be immunosuppressive, either by directly affecting immune mechanisms, or by activating the hypothalamic-pituitary-adrenal (HPA) axis and the sympathetic nervous system [12, 29]. Volatile anesthetics, by mechanisms that are only partially elucidated, impair NK cell, T cell, dendritic cell, neutrophil, and macrophage functions. Furthermore, opioid analgesics were found to inhibit both cellular and humoral immune function in humans [35, 36]. Melamed and colleagues showed that ketamine, thiopental, and halothane, but not propofol, had inhibitory effects on NK cell activity and increased metastatic burden in rats [37]. 

 Opioids suppress the innate and adaptive immune responses [38, 39]. While neural and neuroendocrine responses are also involved [40], the presence of opioid-related receptors on the surface of immune cells increases the likelihood of a direct immune-modulating effect [41]. De Waal and colleagues found different opioids to have differing immunosuppressive effects [42]. Synthetic opioids, however, do not appear to attenuate immune response [43, 44].

 These immunosuppressive factors occur simultaneously during surgery and in the immediate postoperative period. The perioperative period is therefore a decisive period during which interventions that promote host defense may especially benefit the patient [11]. This may be of particular interest in patients undergoing tumor resection. While surgery is essential to reduce tumor burden, and among various treatment options, it is considered to be the most effective treatment for solid tumors; a rapid spread and growth of malignant tissue is often observed after tumor resection [45]. Cancer surgery, even with the best technique, is usually associated with dissemination of malignant cells through the lymphatics and the systemic circulation, and, at the time of surgery, many patients have already established micrometastases [46]. The clinical manifestation of this minimal residual disease is a function of both the host immune competence (particularly NK cell function) and the tumor's proliferative and angiogenic abilities [22, 23, 45, 47]. Regional anesthesia reduces the amount of intraoperative general anesthetics required, has opioid sparing effects, and markedly attenuates the neuroendocrine stress response to surgery as well as preserving NK cell function and T_h_1 cell activity better than general anesthesia [48]. It is hypothesized that regional anesthesia and analgesia help preserve control of tumor progression. Modification to anesthetic management might thus reduce the risk of recurrence [18].

## 6. Imaging Techniques during Epidural Catheterization

Identifying the epidural space and correct needle positioning is often challenging for the novice anesthesiologist. Epidural catheter placement is thought to be among the most difficult techniques to acquire [49], with a success rate of as low as 60% at the first attempt [50], and an overall success rate of nearly 90% [51]. Factors contributing to the success or failure of catheter placement can be surgery related, as the type of surgery determines the specific region of the vertebral column for block placement [52]; patient dependent, such as body habitus, presence or absence of identifiable anatomical landmarks, or spinal anatomy; or operator dependent, such as the degree of personal experience, patient positioning, needle size, or the use of conventional “blind” versus imaging-guided techniques [53]. Previous reports suggest that the conventional “loss of resistance” technique used in the thoracic and lumbar region may have a false-positive success rate of as high as 30%, and, although generally considered reliable for epidural anesthesia, when used as a sole tool, this clinical sign may not offer the same potential to accurately identify the epidural space, as when complemented with an imaging tool [54, 55]. Visualization of the interlaminar space, accurate estimation of the depth to the epidural space, and optimal needle insertion angle are known to facilitate epidural block placement [50, 56, 57]. With the rapidly evolving imaging technology, there has been an increasing interest in the use of various imaging tools, to improve success rates of neuraxial blocks. Several studies have shown the usefulness of both ultrasound guided and fluoroscopically guided catheter insertion techniques [49, 58, 59].

### 6.1. Ultrasound Guided Epidural Catheter Placement

Ultrasound is a radiation-free imaging tool that is now widely used in clinical practice. The first successful sonographic measurement of the epidural space dates back to the 1980s, when Cork and colleagues [60], and Currie [61] were able to localize and estimate the distance from the skin to the epidural space. More recently, Bonazzi and de Gracia identified the ligamentum flavum in the lumbar vertebral region [62]. Technical improvement in sonographic visualization, such as the ability to digitally depict anatomical structures at high resolution, has much increased the clinical feasibility of ultrasound in epidural catheter insertion and visualization [57, 58]. The increasing popularity of this technique over the past three decades has been attributed to a more accurate estimation of epidural space depth, a more optimal determination of the needle insertion point, and insertion angle particularly in cases of difficult anatomy (such as obesity, especially during obstetric anesthesia, or scoliosis), or the presence of implanted hardware [63], and reduced failure rate [56]. While the use of ultrasound offers a greater likelihood of successful catheter placement in the obese patient, morbid obesity may pose technical difficulties to the visualization of the vertebral anatomy and the epidural space.

 Besides the obvious benefits of this radiation-free technique compared to the conventional “blind” method, there are disadvantages of ultrasound use in the setting of epidural block placement. Technically, it can be difficult to simultaneously stabilize and advance the Tuohy needle, and maintain the acoustic window, holding the ultrasound probe in the optimal position. Also, it can be difficult to maintain continuous visualization of the Tuohy needle tip during advancement. The use of ultrasound in adults is helpful for anatomical identification, but there is limited published evidence available for the same degree of usefulness of real time needle insertion, compared to the pediatric population. A recent study by Belavy and colleagues, evaluating the feasibility of real-time 4D ultrasound for epidural catheter placement in cadavers, found that 4D ultrasound potentially improves operator orientation of the vertebral column at the cost of needle visibility and resolution [64–67]. Slight discrepancy between the sonographically and clinically measured epidural space depth should be anticipated, likely due to factors such as tissue deformation during needle passage, deviation from the midline, and deviation from the 90 degree insertion angle that has been found to most precisely correlate with the sonographically measured skin-epidural space distance. When compared to the fluoroscopic visualization, ultrasound guidance does not offer the advantage of placing the epidural catheter exactly at the desired vertebral level; also, the depth of the inserted needle may not always be adequately assessed.

### 6.2. Fluoroscopically Guided Epidural Catheter Placement

The usefulness of fluoroscopic guidance in epidural block placement in various regions of the vertebral column has been established [49, 55, 61]. Previous studies have shown that more than 50% of lumbar epidurals, in the absence of appropriate imaging tools, were actually performed at a level other than the one predicted [68]. A study by Renfrew and colleagues found that caudal blocks without the use of fluoroscopy resulted in a 52% incidence of erroneous needle placement [69], likely due to the subfascial compartment that provides low resistance to injection. Fluoroscopic guidance offers the advantages of precise needle angulation and localization of the catheter at the targeted vertebral level even in the presence of difficult or unreliable surface anatomy, as well as accurate identification of the epidural space, or the assessment of injectate dispersal, with the use of contrast dye to confirm the epidural placement. These factors may also obviate complications [59]. Fluoroscopy therefore improves the success rate of epidural block and provides a reliable delivery of therapeutic substances into the epidural space; however, both the patient and the operator are exposed to radiation. Furthermore, this method may only be safe in patients without contraindication to the use of contrast dye or radiation itself. 

 While the use of imaging tools for epidural catheter placement is gaining increasing popularity for their potential to increase success rate and reduce complications, the potential risks and benefits of these methods should be thoroughly assessed, and the choice of imaging technique should be determined on an individual basis. It should be remembered that the use of ultrasound guidance does not eliminate the need for using the conventional “loss of resistance” technique, and it is as important as when using the blind insertion technique. 

## 7. Considerations in the Pediatric Population

With the development of advanced skills with ultrasound, guided techniques has attracted an increased interest in its use for neuraxial blocks. The benefits of identifying anatomy and directly visualizing needles and catheters, as found with peripheral blocks, can be of great value for improved success and confirmation of neuraxial blocks. Because of the large variation of each patient's body habitus due to age, it can be difficult to predict the puncture depth to reach either the epidural or intrathecal spaces [70]. 

 In pediatric population, checking the anatomy with the ultrasound before and during performing the procedure gains and assures a lot of success. Visualization is clearer than in the adult population due to less ossification of the vertebral column and easiness to predict the epidural and/or the intrathecal spaces. Loss-of-resistance technique to identify the epidural space can be very challenging in neonates due to presence of less fibrous tissue limiting the tactile feedback [71]. 

 Visibility of the spread of fluid is a promising technique during injection through the needle and catheter, which could confirm the position. Using an epidural electrical stimulation test is another method but the clinical value of electrical stimulation in caudal needle placement has not been extensively studied [72]. 

### 7.1. Caudal Needle and Catheter Placement under Ultrasound

**Figure 1:**
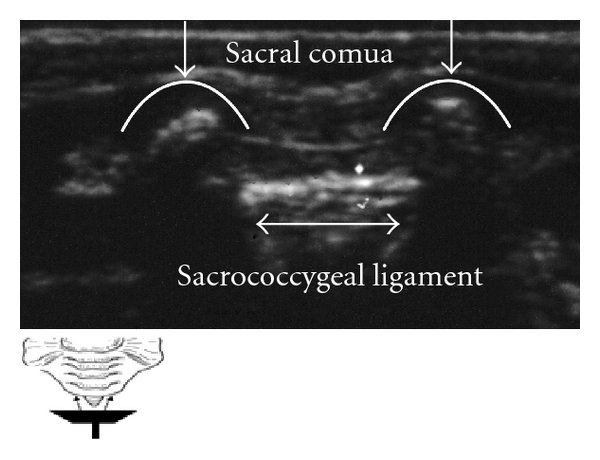
Placing the probe transverse plane at the coccyx, the sacral cornua (represented in white arrows heading down) are viewed laterally as humps. Sacral hiatus is located between an upper hyperechoic line, representing the sacrococcygeal membrane or ligament and an inferior hyperechoic line representing the dorsum of the pelvic surface of the sacrum (bidirectional sided arrow).

**Figure 2:**
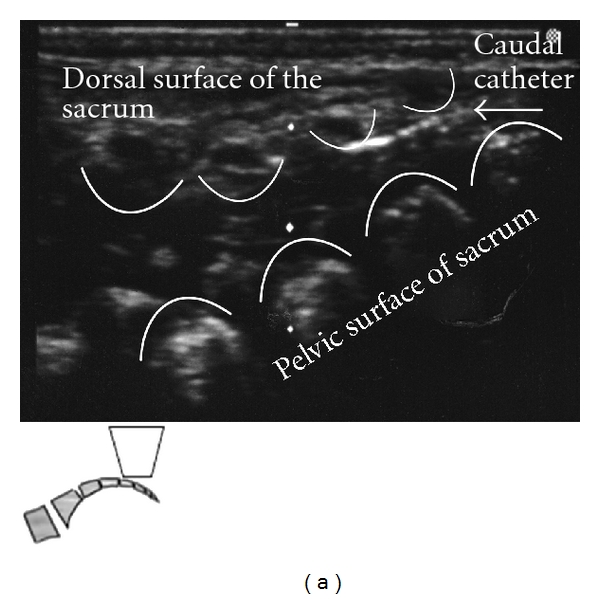
(a) Placing the probe longitudinally between the sacral cornua will capture the dorsal surface of the sacrum, the dorsal aspect of the pelvic surface of the sacrum, and the sacrococcygeal ligament. Angiocatheter penetrated the sacrococcygeal ligament and lies in the epidural space. (b) Local Anesthetic spread through Caudal Angiocatheter in caudal epidural space.

**Figure 3:**
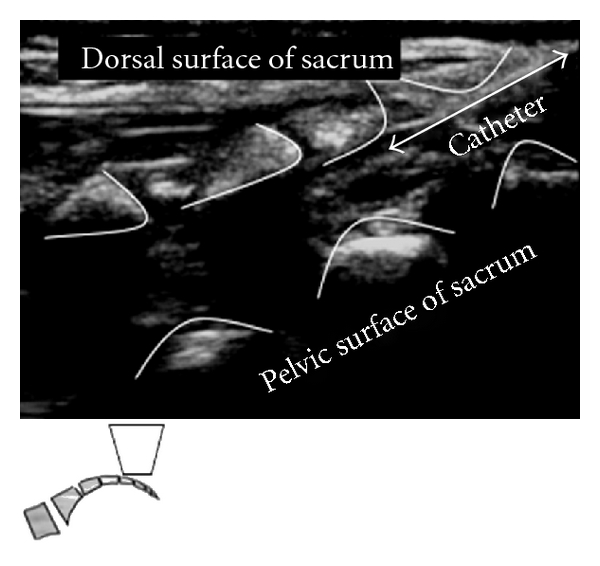
Caudal epidural catheter passing through Angiocatheter in the epidural space.

**Figure 4:**
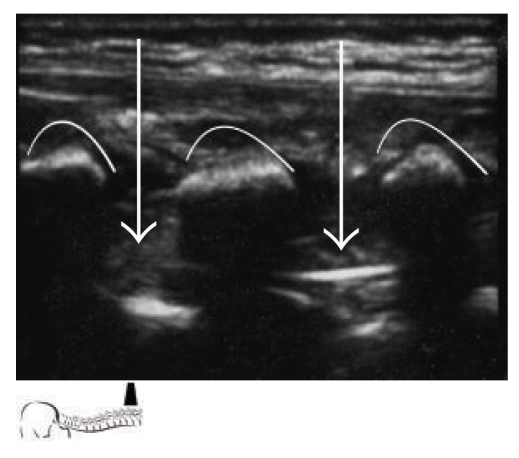
Longitudinal view at the thoracic spine level, viewing the advancement of the caudal epidural catheter. Curved lines show spinous processes. Arrows show epidural catheter in between spinous processes.

**Figure 5:**
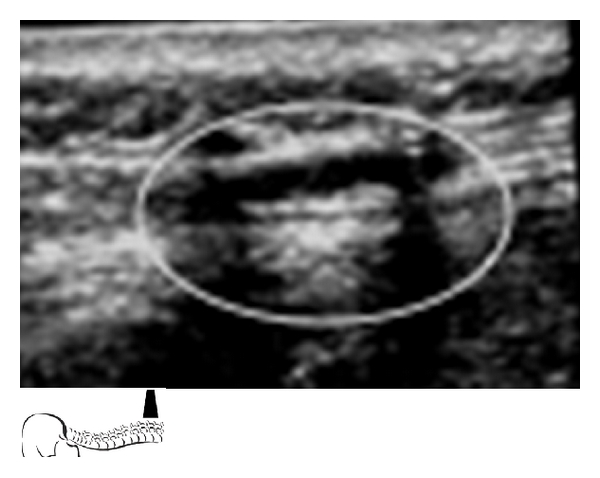
Longitudinal view at the lumbar spine. Visualization of the local anesthetic spread confirmed the position.

Caudal anesthesia is one of the most popular regional blocks in the pediatric population to provide perioperative analgesia. Placement of a single shot caudal block or a lumbar/thoracic epidural catheter achieved through the caudal epidural space is an advanced skill. This technique becomes even more complex when considering variation in patient age, weight, and varying levels of bone ossification. Ultrasound guidance for this procedure is helpful in identifying the underlying anatomic structures. The ones most commonly of interest include the sacral hiatus, sacral cornua, coccyx, and sacrococcygeal ligament. While probe orientation can be done using either a transverse or longitudinal view of the midline, it is typically best to orient and assess landmarks prior to performing the procedure (Figures 1, 2, 3, 4, and 5).

 When introducing a catheter into the caudal space to reach the lumbar or thoracic spine, a technique similar to the above is used for cannula placement. The catheter can then be directly visualized during advancement with the ultrasound at each level of the spine above the sacrum (Figures 2(a), 2(b), and 3).

As is the case during the assessment, either the longitudinal or transverse axes can be used to visualize the underlying structures and catheter position. 

Confirmation of catheter placement can be performed through visualization of local anesthetic spread as well as through direct visualization of the catheter within the epidural space. Catheter tip visibility may be improved with the injection of a bubble-based fluid or local anesthetic spread and a swoosh test (using a stethoscope to listen to fluid movement).

### 7.2. Tunneling of Caudal Epidural Catheter

Bacterial colonization is regarded as a causative factor for infectious complications of caudal catheters in children [73]. In addition to the routine measures of wearing personal protective equipment (hats, masks, and gloves), prepping the area with an alcohol-based solution, and maintaining a sterile field, another option is to tunnel the catheter after placement. A small subcutaneous placement of the proximal portion of the catheter not only decreases the length of tubing potentially exposed to contamination, but it also helps in gaining a more secure catheter placement. Both of these features become especially advantageous in prolonged epidural catheter use.

## 8. Complications of Epidural Anesthesia 

Epidural anesthesia and analgesia are generally considered to be safe with regards to adverse post procedural events, as their complications, resulting in permanent deficits, are rare. Besides their indications and obvious benefits, knowledge of adverse outcomes should also comprise an essential part of clinical decision making. 

 Complications of central neuraxial blockade, much depending on the experience in patient management, as well as materials, equipment, and the presence of risk factors, have been reported to occur at various frequencies [74, 75]. An epidemiologic study conducted in Sweden over a period of 10 years revealed an increasing trend (1 in 10,000 neuraxial anesthetics) of severe complications after central neuraxial blockade [74]. Relatively recent literature suggests that most of these occur with the perioperative use of epidural block [74, 76]. The incidence of major complications (permanent harm including death) of epidural and combined spinal-epidural anesthesia were at least twice as high as those of spinal and caudal blocks, as reported by Cook and colleagues. This study also found that the incidence of epidural catheter-related serious morbidity and mortality was higher when blocks were placed in the perioperative setting, as opposed to catheter placement in obstetric and pediatric populations, when inserted for chronic pain management, or when placed by non-anesthetists [77]. While prognosis is infrequently reported, retrospective reviews report full recovery in 61–75% of patients, epidural hematoma accounting for two-thirds of residual neurological deficits [77, 78]. Serious complications, if not recognized and treated at an early stage, may thus result in permanent loss of function [74, 79]. With regards to the timing of catheter placement, there is still substantial controversy: while many anesthesia providers believe that epidural catheters should be placed in awake or mildly sedated patients capable of providing feedback [80], Horlocker's retrospective review found no evidence of an increased risk for neural injury in anesthetized patients receiving epidural anesthetic [81]. Thoracic epidural placement, however, should never be attempted on an anesthetized patient. Having increasingly become the focus of attention, and as a result of both meticulous adherence to sterile, atraumatic catheter insertion technique and management, as well as careful risk-benefit assessment, major complications of epidural anesthesia are now rare, particularly those not involving infection or bleeding, and many resolving within 6 months [74]. The estimation of the incidence of all adverse outcomes, however, is often inaccurate.

 Complications may occur early if related to traumatic catheter insertion, or later in the operative-postoperative course if caused by catheter-related spinal space-occupying lesions such as epidural hematoma or abscess formation, and are infrequent among the general population. Although its incidence is lower than when associated with spinal anesthesia [80], transient neurological injury has been found to account for the majority of short-term epidural catheter related complications (1 in 6,700) in a meta-analysis by Ruppen and colleagues, followed by deep epidural infections (1 in 145,000), epidural hematoma (1 in 150,000–168,000), and persistent neurological injury (1 in 257,000) in women receiving epidural catheter for childbirth [82, 83]. Spinal epidural hematoma, however, has been recently suggested to occur in a rate as high as 1 in 3,600 in female patients undergoing knee arthroplasty [74, 84, 85]. These findings were consistent with those previously reported in the ASA Closed Claims Project database analysis by Lee et al; however, limitations of that study design and database do not allow risk quantification specific to regional anesthetic techniques or populations [86].

 Adverse events may result from direct mechanical injury or adverse physiological responses. Neurological complications resulting from accidental penetration of the dura are similar to those that occur with spinal anesthesia. Inadvertent dural puncture and postdural puncture headache, direct neural injury, total spinal anesthesia, and subdural block have been commonly reported. The incidence of inadvertent dural puncture ranges between 0.19–0.5% of epidural catheter placements. Postdural puncture headache (PDPH), described as a positional, bilateral frontal-occipital, nonthrobbing pain, may develop in as much as 75% of patients [87–89]. PDPH is thought to develop as a result of persistent transdural leakage of cerebrospinal fluid (CSF) at a rate that is faster than that of CSF production. The subsequently decreasing CSF volume and pressure causes traction on the meninges and intracranial vessels, which refer pain to the frontal-occipital region, often extending to the neck and shoulders, more pronounced in the upright position. Available measures of prevention besides conservative measures are immediate intrathecal catheter placement, prophylactic epidural blood patch, epidural or intrathecal administration of saline, and epidural administration of morphine [90]. Direct neural injury has a reported incidence of 0.006% [82], and has been associated with paresthesias during needle placement and pain on injection [80]. Total spinal anesthesia may occur if the solution used for epidural anesthesia is inadvertently administered into the intrathecal space in large volumes. Symptoms are of a rapidly arising subarachnoid block, potentially resulting in cardiovascular collapse and apnea requiring prompt resuscitation. Provided that immediate, skilled resuscitative efforts are made, complete recovery should be expected [91]. While clinically not always distinguishable from epidural blocks, the incidence of clinically recognized subdural block was found to be 0.024% in a prospective study [92]. A subdural block may present as high sensory block, often with sparing of motor and sympathetic fibers, is slow in onset, and the blockade is disproportionately extensive for the volume of anesthetic injected. Clinical signs and symptoms may be mistaken for accidental intrathecal injection, migration of epidural catheter, or an asymmetrical, patchy or inadequate epidural block. Subdural placement is thought to occur independently of the operator's expertise. Although there are no established risk factors, recent lumbar puncture and rotation of the needle may predispose to subdural injection [93].

 Hemorrhagic complications are serious adverse outcomes that may arise from neuraxial anesthesia. Epidural hematoma is a rare, but potentially devastating, complication that requires emergency decompression in case of clinical deterioration. It is rarely attributed to an arterial source, and can develop spontaneously [94, 95]. While paralysis may occur even after hematoma evacuation, it is still not precisely understood why several of the spinal epidural hematomas associated with concurrent anticoagulant use involving less blood than the volume injected when performing a therapeutic blood patch [85]. Clinically significant bleeding is more likely with congenital or acquired coagulation abnormalities, thrombocytopenia, vascular anomalies or anatomical abnormalities, advanced age and female gender, repetitive attempts at catheter insertion, and traumatic block placement [74, 96–98]. The risk is reported to increase 15-fold when there is a concomitant use of anticoagulants, and appropriate precautions are not taken [85]. Appropriate timing of anticoagulant administration is important in decreasing the risk of bleeding [99]. The commonest presenting symptoms of spinal epidural hematoma are new back pain, radicular pain, and progressive lower extremity weakness. Symptoms rarely present immediately after surgery, but may develop while the catheter is still in place. These symptoms can occur 15 hours to 3 days after catheter insertion [78, 98]. The diagnostic investigation of choice is MRI. A delay in diagnostic imaging may lead to devastating outcomes, and is a common error, as manifesting neurological symptoms and back pain may be attributed to the use of epidural infusion and a prolonged effect of local anesthetic, and to musculoskeletal origin [78, 100]. Cauda equina syndrome due to hematoma formation, a rare complication with a reported incidence of 2.7/100,000 epidural blocks, was found to result in permanent deficit in more than two-third of the cases [74]. Classic manifestation is low back pain, altered proprioception and decreased sensation to pinprick and temperature in the lumbar and sacral nerve distribution, voiding and defecation disturbances, and progressive loss of muscle strength. Outcomes are primarily function of interval to hematoma evacuation and the severity of the neurological deficit, and are favorable if decompression is performed within 8 hours of the development of symptoms [98]. 

 Epidural catheter related infections are rare complications both in adult and in pediatric patients. A retrospective database analysis by Sethna et al. found an expected incidence ranging between 3–13/10,000 catheters in children [101]. Epidural abscess and meningitis has been reported to occur in 1 : 1000 and 1 : 50,000 catheter placements, respectively [74]. Although epidural catheters are placed under aseptic conditions, needle or catheter contamination does occur even during aseptic puncture and sterile handling of devices [102]. Of patient risk factors, skin colonization at the puncture site and bacterial migration along the catheter is proposed to be the most likely route of infection; however, immunosuppression [74, 103], diabetes mellitus [104], chronic renal failure, steroid administration, cancer, herpes zoster, rheumatoid arthritis [105], systemic or local sepsis, and prolonged infusion duration are also identifiable risk factors. The rate of skin colonization at puncture sites is reported to be higher in children than in adults, with an overall incidence as high as 35% [101]. The incidence of infection increases after three days [106]. The classic presentation signs and symptoms are severe midline back pain, fever, and leukocytosis, with or without neurological symptoms (worsening lower limb weakness and paraplegia, incontinence, irradiating pain, nuchal rigidity, and headache). Symptoms commonly appear after removal of the epidural catheter [78]. Neurological deficits have been found to be persistent in more than 50% of patients developing epidural abscess [105]. Barrier precautions, skin disinfection [107], as well as the use of closed epidural system, and patient-controlled epidural analgesia [101] have been suggested as ways to decrease the incidence of epidural catheter-associated infections. Frequent syringe changes, on the other hand, may be associated with a higher rate of epidural infections [108]. Frequently implicated infecting organisms are Methicillin-resistant Staphylococcus aureus (MRSA), Staphylococcus aureus, and Coagulase-negative Staphylococcus [101, 109]. Outcomes are favorable when diagnosed and treated promptly. Adhesive arachnoiditis, presenting in various forms, is a sterile inflammatory response to accidental subarachnoid injection of local anesthetics, preservatives, detergents, or antiseptics [110–112], and has also resulted from traumatic puncture or epidural abscess. Medical literature suggests an extremely low incidence [113, 114].

Complications of epidural anesthesia are rare events that may result in detrimental sequelae. Strict adherence to prophylactic measures and treatment without delay is essential to further lower the incidence of adverse outcomes.

## 9. Epidural Anesthesia and Thromboprophylaxis

Some controversy exists with regards to reduced coagulation and neuraxial anesthesia and challenges are emerging as new agents are introduced into clinical practice. Spinal epidural hematoma, although still considered to be a rare complication occurring at a previously reported rate of less than 1 in 150,000 epidural and less than 1 in 220,000 spinal anesthetics in patients with normal coagulation status, is now suggested to occur in a rate as high as 1 in 3,000 in some patient populations [84, 85]. Patients receiving antithrombotic or antiplatelet therapies are more at risk for this potentially dramatic adverse event, in particular after invasive procedures [98]. In the United States, the estimated incidence of spinal epidural hematoma with concurrent administration of antithrombotic drugs (low molecular weight heparins) is 1 : 40,800 for spinal anesthesia, 1 : 6,600 for single-shot epidural anesthesia, and 1 : 3,100 for continuous epidural anesthesia [85]. Risk factors for epidural bleeding were established as coagulation disorders, antithrombotic or fibrinolytic therapy, or the use of any agents interfering with coagulation, female gender, age, difficult vertebral or spinal cord anatomy, difficult or traumatic catheter insertion, and lack of guidelines [96, 98, 115, 116]. Catheter removal carries nearly the same risk as insertion [98]. Appropriate time intervals between the administration of anticoagulants, neuraxial block placement, and catheter removal are crucial in the prevention of hematoma formation [117, 118].

The American Society of Regional Anesthesia and Pain Medicine (ASRA), and more recently, the European Society of Anaesthesiology (ESA) published their consensus statements on neuraxial anesthesia and the use of antithrombotic and thrombolytic agents [99, 119]. While providing guidelines in clinical decision making, and having the aim of minimizing hemorrhagic complications, these recommendations do not guarantee a specific outcome, and allow of variations based on the judgment of the anesthesiologist. The guidelines of the American Society of Regional Anesthesia and Pain Medicine and the European Society of Anaesthesiology are based on previously published national recommendations, hematology, pharmacology, and risk factors for surgical bleeding, and incorporate updated information since the time of their publication. 

 With regards to epidural catheter placement, the ASRA recommends that patients receiving thrombolytic therapy be queried and their medical records reviewed for a recent history of lumbar puncture or neuraxial analgesia. Neuraxial anesthesia should be avoided, or, if received concurrently with the fibrinolytic/thrombolytic therapy, close neurological monitoring should be continued along with the administration of neuraxial solutions that minimize sensory and motor block. There is no definitive recommendation for epidural catheter removal in patients receiving fibrinolytic and thrombolytic therapy. Thrombolytics, if scheduled, should be avoided for 10 days after puncture of noncompressible vessels [99]. 

 Patients receiving unfractionated heparin (UFH) thrice a day, if recommended by recent thromboprophylaxis guidelines, may be at an increased risk of surgical-related bleeding. The ASRA recommends that the patient's—potentially simultaneous—anticoagulant and antiplatelet medication be daily reviewed. There is no contraindication to epidural blockade in patients receiving subcutaneous UFH prophylaxis at daily doses of 2 × 5000 U. The risk of bleeding may be increased in debilitated patients receiving prolonged therapy, and may be decreased by delaying the heparin injection until after neuraxial block placement. The safety of central neural block in patients receiving subcutaneous UFH in a dosing regimen of more than 10,000 U daily has not been established, and an increased risk of a spinal epidural hematoma has also not been elucidated. Patients receiving heparin for greater than 4 days should be assessed for heparin-induced thrombocytopenia (HIT). In patients with known coagulopathies, combining neuraxial techniques with intraoperative heparinization should be avoided however, this technique is acceptable in patients with no other coagulation disorders, if

1. heparin administration is delayed for 1 hour after puncture,
2. epidural catheters are removed 2 to 4 hours after the last heparin dose and the patient's coagulation status is assessed. the next heparin dose may be administered 1 hour after catheter removal,
3. patient is closely monitored for early signs of neurologic dysfunction while receiving neuraxial solutions that minimize sensory and motor block postoperatively.

Per the ASRA guidelines, in contrast with the ESA recommendations that suggest considering postponement of the procedure, difficult or traumatic block placement should not necessarily prompt postponing surgery; however, the potential benefits should be carefully weighed against all potentially detrimental outcomes in each individual. With regards to the full anticoagulation of patients undergoing cardiac surgery, the ASRA finds insufficient evidence available to determine an increased risk of neuraxial hematoma. Close postoperative monitoring of neurologic function, as well as administration of neuraxial solutions that minimize sensory and motor block to facilitate detection of signs and symptoms of cord compression, is however suggested [99, 119].

 Patients on low molecular weight heparin (LMWH) anticoagulation have not been found to be at an increased risk of bleeding in high-risk groups, contrasting with patients receiving UFH-thromboprophylaxis. Also, compared to UFH, LMWH-therapy has been associated with a significant decrease in the risk of HIT, as demonstrated by Warkentin and colleagues [120]; nonetheless, LMWHs are contraindicated in such condition due to the high level of cross-reactivity. To avoid an elevated risk of bleeding complications, an interval of 10 to 12 hours between preoperative LMWH administration at prophylactic doses and needle placement or catheter removal is recommended. Administration of LMWH the night before surgery does not thus interfere with epidural block placement on the day of surgery. In patients on therapeutic doses of LMWH, catheter placement should be delayed for a minimum of 24 hours after the last dose. Patients undergoing general surgery and receiving LMWH 2 hours prior to surgery are not ideal candidates for a neuraxial blockade, and are thus recommended against neuraxial techniques. Patients receiving postoperative LMWH thromboprophylaxis may safely be administered both single-dose and continuous catheter techniques. With regards to management, timing of the first postoperative dose, dosing schedule, and total daily dose are authoritative. Concerning the management of patients receiving LMWH, the ASRA recommends against the routine monitoring of anti-Xa level and concurrent administration of medication affecting hemostasis, regardless of LMWH dosing regimen [99, 119].

 The management of patients receiving perioperative oral anticoagulants is still controversial. In the United States, much like in Europe, therapeutic oral anticoagulation is considered as a contraindication to central neuraxial blockade. As opposed to Europe, however, perioperative thromboprophylaxis is still possible in the United States. According to the recommendation of the American Society of Regional Anesthesia and Pain Medicine, warfarin therapy must be stopped ideally 4-5 days before the scheduled procedure, and the INR checked before neuraxial block placement. In patients receiving an initial preoperative dose of warfarin, INR should be measured before needle puncture if the administration of the first dose exceeded 24 hours, or if a second dose of such anticoagulant has been administered. In patients at risk for an enhanced response to oral anticoagulants, a reduced dose of drug should be administered. In patients receiving low-dose warfarin during epidural analgesia, INR should be monitored daily. Epidural catheters should be removed when the INR is less than 1.5. If the INR is greater than 1.5 but less than 3, indwelling epidural catheters should be done with caution. The ASRA recommends against concurrent use of agents, such as UFH, LMWH, or platelet aggregation inhibitors, that influence other components of the clotting system, as these, without affecting the INR, may increase the risk of bleeding. Medical records should be reviewed for such agents. Neurologic testing of sensory and motor function should be performed routinely during epidural analgesia for patients on oral anticoagulants, and should be continued for at least 24 hours after catheter removal, until the INR returns to the desired prophylactic range. In patients with INR greater than 3, the American Society of Regional Anesthesia and Pain Medicine recommends that the warfarin be held or reduced, without making a definitive recommendation regarding the management to facilitate catheter removal in these patients [99, 119].

 Platelet aggregation inhibitors, such as acetylsalicylic acid, thienopyridines (clopidogrel, ticlopidine, and prasugrel), glycoprotein (GP) IIb/IIIa antagonists (eptifibatide, tirofiban, and abciximab), the novel ADP P2Y12 receptor antagonist ticagrelor, and the selective phosphodiesterase IIIA inhibitor cilostazol, have diverse effects on platelet function. No wholly accepted test exists to guide antiplatelet therapy. It is therefore critical to perform a careful preoperative risk assessment to identify factors that might potentially contribute to bleeding. Although administration of nonsteroidal anti-inflammatory drugs (including aspirin) does not appear to significantly increase the incidence of hematoma formation, concurrent administration of LMWH, UFH, or oral anticoagulants resulted in a higher rate of complications in both surgical and medical patients, their use along with NSAIDs, including aspirin, is therefore not recommended. Cyclooxygenase-2 inhibitors have minimal inhibitory effect on platelet aggregation, and should be considered in patients requiring anti-inflammatory therapy in the presence of anticoagulation. The actual incidence of spinal epidural hematoma related to thienopyridines and GP IIb/IIIa inhibitors is not known. Management should be based on labeling precautions and the experience of professionals involved in the clinical care of the patient. However, as it has been suggested by recent guidelines, ticlopidine and clopidogrel therapy should be discontinued 14 and 7days prior to neuraxial block, respectively. If needle puncture is indicated between 5 and 7 days of discontinuation of clopidogrel, normalization of platelet function should be documented. GP IIb/IIIa antagonists exert a dose-dependent effect on platelet aggregation. After the last administered dose, the time to normal aggregation is 4 to 8 hours for eptifibatide and tirofiban, and 24 to 48 hours for abciximab. Neuraxial blockade should be avoided until normal platelet function is achieved. Should a patient, despite the contraindication, be administered GP IIb/IIIa inhibitors within 4 weeks of surgery, careful neurological monitoring should be performed [99, 119].

 Both the ASRA and the ESA guidelines recommend against the mandatory discontinuation of herbal agents (most commonly: garlic, Echinacea, Gingko biloba, ginseng, aloe vera, and ephedra of dwarf palm), neither should neuraxial techniques be avoided, as there is insufficient evidence that these, by themselves, significantly increase the risk for spinal hematoma formation. There is insufficient evidence to conclude that thrombin inhibitors, such as lepirudin, desirudin, bivalirudin, or argatroban, are safer to use in patients receiving spinal or epidural anesthesia; performance of these techniques in the presence of these agents is thus not recommended. Until sufficient evidence is available, neuraxial techniques in patients receiving fondaparinux should only be performed if single needle pass, atraumatic block placement, and avoidance of indwelling catheters are feasible, or a different method of prophylaxis should be considered [99, 119].

## 10. Summary

Epidural and caudal anesthesia is a versatile neuraxial anesthetic technique with an expanding area of indication. It can be used in the perioperative setting as the sole anesthetic, or in combination with general or spinal anesthesia. Its potential to decrease postoperative complication rate by its beneficial physiological effects has been clearly demonstrated in several studies. The absolute contraindications to its use have traditionally been well defined. Despite its rare, but potentially devastating complications, neuraxial anesthesia is considered to be safe. Performing such procedures in the presence of anticoagulants is however controversial. With patients presenting with medical conditions that predispose to clinically significant bleeding and an increased number of patients taking various anticoagulants, there is greater concern for an increased incidence of epidural hematomas. The key to maximizing the advantages while minimizing the disadvantages of epidural and caudal anesthesia is to become familiar with the anatomical, physiological, pharmacological, and technical aspects of block placement. The review and advances discussed here allow both adult and pediatric populations a form of care that is often considered indispensable.
